# Compact hard X-ray split-and-delay line for studying ultrafast dynamics at free-electron laser sources^1^

**DOI:** 10.1107/S1600577519004570

**Published:** 2019-06-04

**Authors:** Rustam Rysov, Wojciech Roseker, Michael Walther, Gerhard Grübel

**Affiliations:** a Deutsches Electronen Synchrotron, Notkestrasse 85, 22607 Hamburg, Germany; b Centre for Ultrafast Imaging, Luruper Chaussee 149, 22607 Hamburg, Germany

**Keywords:** X-ray optics, delay lines, free-electron lasers, FELs, ultrafast dynamics, X-ray photon correlation spectroscopy, XPCS, pump–probe experiments

## Abstract

A compact hard X-ray split-and-delay line for studying ultrafast dynamics at free-electron laser sources is presented. The device is capable of splitting a single X-ray pulse into two fractions to introduce time delays from −5 to 815 ps with femtosecond resolution. The split-and-delay line can operate in a wide and continuous energy range between 7 and 16 keV. Compact dimensions of 60 × 60 × 30 cm with a total weight of about 60 kg make it portable and suitable for direct installation in an experimental hutch. The concept of the device is based on crystal diffraction. The piezo-driven stages utilized in the device give nanometre positioning accuracy. On-line monitoring systems based on X-ray cameras and intensity monitors are implemented to provide active alignment feedback. Performance estimates of the system are also presented.

## Abstract 2

Presented here is a compact hard X-ray split-and-delay line for studying ultrafast dynamics at free-electron laser sources. The device is capable of splitting a single X-ray pulse into two fractions with delay times from −5 to 815 ps and femtosecond resolution, operating continuously in a wide energy range between 7 and 16 keV.

## 1. Introduction   

Hard X-ray free-electron laser (FEL) sources based on self-amplified spontaneous emission (SASE) provide spatially coherent ultrashort pulses with extremely high peak brightness (Emma *et al.*, 2010 ▸; Ishikawa *et al.*, 2012 ▸; Tschentscher *et al.*, 2017 ▸; Kang *et al.*, 2017 ▸). Such superior beam properties provide excellent conditions for conducting ultrafast dynamics studies, for instance initiated by an optical or X-ray pump pulse (Glownia *et al.*, 2010 ▸; Trigo *et al.*, 2013 ▸, 2008 ▸) or via X-ray photon correlation spectroscopy (XPCS) (Grübel *et al.*, 2007 ▸; Grübel & Zontone, 2004 ▸; Lehmkühler *et al.*, 2015 ▸, 2018 ▸). However, the repetition rates of FEL pulses are mostly limited to tens of hertz (Emma *et al.*, 2010 ▸; Ishikawa *et al.*, 2012 ▸) or 4.5 MHz (Tschentscher *et al.*, 2017 ▸). In order to access timescales faster than provided by the intra-bunch spacing of FEL sources, one can employ accelerator-based double-pulse technology (Hara *et al.*, 2013 ▸; Sun *et al.*, 2017 ▸; Marinelli *et al.*, 2015 ▸) or optical split-and-delay lines.

In the past decade, several hard X-ray split-and-delay systems have been developed. The first device of this kind was manufactured at DESY. It employed amplitude division splitting by thin silicon crystals and fixed scattering-angle geometry (Roseker *et al.*, 2009 ▸). The performance was successfully verified at storage rings (Roseker *et al.*, 2011 ▸) and FEL sources (Roseker *et al.*, 2012 ▸) by demonstrating the first ultrafast XPCS study of nanosecond colloidal dynamics (Roseker *et al.*, 2018 ▸). Following this work, new split-and-delay devices have been developed at the LCLS, USA (Zhu *et al.*, 2017 ▸), SACLA, Japan (Osaka *et al.*, 2014 ▸, 2016 ▸; Hirano *et al.*, 2018 ▸) and the European XFEL, Germany (Lu *et al.*, 2018 ▸). However, all of them are integrated into the optical infrastructure and designed specifically for a single FEL instrument.

In this paper we present a compact, versatile and portable split-and-delay device dedicated to conducting X-ray pump–probe and XPCS experiments at any FEL source.

## 2. Basic concept and design   

**Figure 1:**
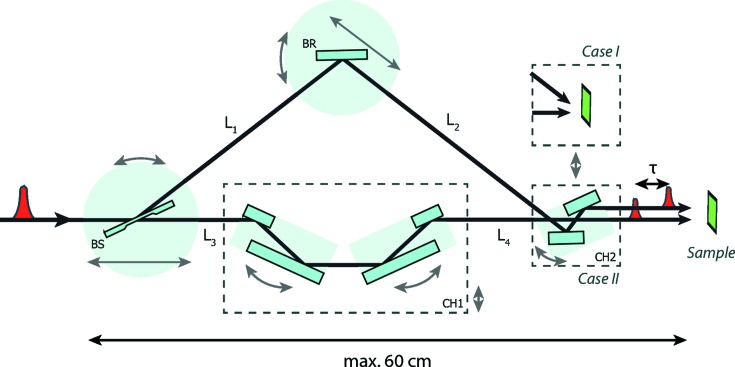
Basic concept of the compact split-and-delay line. Arrows next to the components denote the main crystal motions. CH1 denotes the double channel-cut crystal system. The CH2 module changes the directions of the output beams, as shown in Case I and Case II.

The concept of the split-and-delay line is based on Bragg crystal optics. Fig. 1 ▸ shows a schematic view of the concept. A single FEL pulse is divided by the beam splitter (BS) into two fractions. The first part follows a fixed-delay branch defined by a pair of channel-cut crystals CH1. The path of the second pulse is defined by the variable-delay branch and is guided by the Bragg crystal (BR) and further through the CH2 module. The device is designed to be placed in the experimental hutch near the sample stage or with the sample stage inside the setup, as shown by Case I in Fig. 1 ▸.

Both pulses travel in the sample direction with a time delay τ that is given by
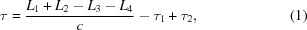
where *L*_1_, *L*_2_, *L*_3_ and *L*_4_ are the corresponding distances between the crystals. The delay times provided by the CH1 and CH2 channel-cut crystals are denoted τ_1_ and τ_2_, respectively. The crystals BS and BR are mounted on movable stages and their relative positions define the delay time between the exit pulses. The CH1 module is used to extend the path length of the fixed-delay branch and provides a possibility of ‘zero delay’ between the pulses (*i.e.* two pulses arrive at the sample simultaneously). The CH1 module can also be translated out of the beam path. The CH2 module, mounted at the exit of the device, consists of two crystals and is used to control the angle between the exit beams. The presented scheme can operate in both horizontal and vertical scattering geometry. The compact design of the device gives the possibility of mounting it in an experimental hutch. In this case, the distance to the sample is greatly reduced, allowing the minimization of any angular instabilities of the beam caused by upstream optical components.

## 3. Crystal optics   

Perfect Bragg crystals are implemented in the design to split and reflect the X-ray beam inside the device. The high diffraction angles achievable with crystal optics can provide higher delay times and small setup dimensions compared with X-ray mirrors (Wöstmann *et al.*, 2013 ▸). Silicon was chosen as the crystal material because of its well known favourable processing properties. Bragg crystals Si(111), Si(220) and Si(422) are used in various combinations. To achieve a geometric intersection between the two beam parts, the crystal BR always has a higher reflection order than the crystal BS. Sets of Bragg crystals are implemented in the following configurations. Si(111)–Si(220) and Si(220)–Si(422) are used as BS and BR crystals, respectively. For the CH1 module, a pair of Si(220) channel-cut crystals is implemented. They have a 10 mm channel-cut gap and are mounted on separate goniometer stages. In addition, a crystal set CH2 is installed in the variable-delay branch (see Case II in Fig. 1 ▸) to obtain parallel output beams. The offset between the beams can be aligned by moving and rotating the CH2 module independently.

Within the energy range from 7 to 16 keV, the Bragg optics covers a range of reflection angles from 7 to 53°. This gives a path-length difference of up to 244 mm and corresponds to a time delay of 815 ps. Perfect single crystals of Si(111) and Si(220) provide 96% and 98% peak reflectivity, respectively, in the aforementioned energy range.

Amplitude division is a default method used to divide the incoming pulse into two fractions, preserving its size and shape. It is achieved by using a thin (<20 µm) Bragg crystal to diffract part of the beam to a variable-delay branch and transmit the remaining part to the fixed-delay branch. The reflected part of the beam is defined by the X-ray photons with energies within the Bragg bandwidth of the beam-splitting crystal. The rest of the beam corresponds to X-ray energies outside the Bragg bandwidth.

The beam splitters were fabricated at Osaka University (Osaka *et al.*, 2013 ▸) using plasma chemical vaporization machining (PCVM) (Mori *et al.*, 2000 ▸). The implemented beam splitters have a very high crystalline quality (crystal roughness < 0.2 nm r.m.s.) with a sufficiently thin (<15 µm) splitting area. An advanced clamping mechanism was developed for the beam splitters. It minimizes crystal lattice strain caused by physical pressure of the holder on the crystal. The high crystalline perfection of the crystals was verified by topography measurements.

Wavefront division can be optionally employed as a splitting mechanism. It can be achieved by simply inserting a sharply polished reflective crystal edge into the incident beam (Hirano *et al.*, 2018 ▸; Osaka *et al.*, 2017 ▸; Zhu *et al.*, 2017 ▸).

## 4. Performance estimates   

**Figure 2:**
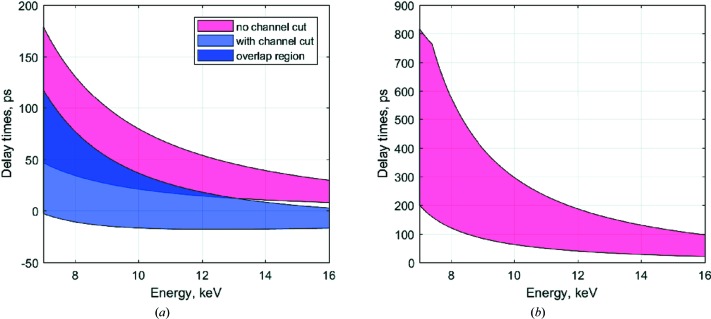
Achievable delay times (*a*) with Si(111)–Si(220) crystals and an Si(220) channel-cut crystal, and (*b*) with Si(220)–Si(422) crystals, in the energy range from 7 to 16 keV. The purple region is accessible in both crystal configurations.

The split-and-delay line can introduce continuous time delays from −5 to 815 ps. A short time delay from −5 up to 179 ps is available with the Si(111)–Si(220) crystal configuration and using the CH1 and CH2 modules. Ultrafast dynamics in electronic structure and the response of various systems can be studied in this regime. Longer delay times up to 0.8 ns can be achieved without channel-cut CH1 and using a two-crystal configuration of Si(220)–Si(422) optics. Fig. 2 ▸(*a*) shows the calculated delay range achievable using Si(111)–Si(220) crystal optics for the BS and BR crystals, respectively. By inserting Si(220) channel-cut crystals in the fixed-delay branch, the achievable delay range can be extended, as shown by the blue shaded area. Fig. 2 ▸(*b*) shows the achievable delay range with the use of Si(220)–Si(422) optics. Using this configuration, a delay time of up to 815 ps can be achieved without using a channel-cut CH1 in the fixed-delay branch.

Table 1 ▸ summarizes available delay times and the overall throughput of the split-and-delay line. The overall throughput for the two-crystal system [*e.g.* Si(111)–Si(220)] can be described as
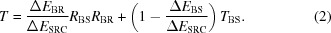
Here, Δ*E*_BS_, Δ*E*_BR_ and Δ*E*_SRC_ are the energy resolutions of the beam splitter BS, crystal BR and the source, respectively. For Si(111) and Si(220) the energy resolution Δ*E*/*E* equals 1.4 × 10^−4^ and 6.04 × 10^−5^, respectively. *R*_BS_ and *R*_BR_ correspond to the crystal reflectivity of the beam splitter BS and beam reflector BR, respectively. *T*_BS_ denotes a transmission factor of the beam splitter.

The total throughput is described by equation (2) for the fixed-delay and variable-delay branches, respectively. In the case of using CH1 and CH2 modules, additional components should be included:
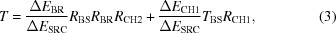
where *R*_CH1_ and *R*_CH2_ are the total throughput of the CH1 and CH2 modules, respectively.

As shown in Table 1 ▸, time delays from 47 to 179 ps can be achieved using only the Si(111) and Si(220) combination for BS and BR, respectively, providing 91% of the incident flux. As an additional crystal component, the CH1 and CH2 modules can be used in combination with BS and BR to reach the near-zero delay time range. A channel-cut CH1 is employed in the fixed-delay branch (see Fig. 1 ▸) to reach time delays from −5 ps using a pair of Si(220) channel-cut crystals.

## 5. Mechanical design   

**Figure 3:**
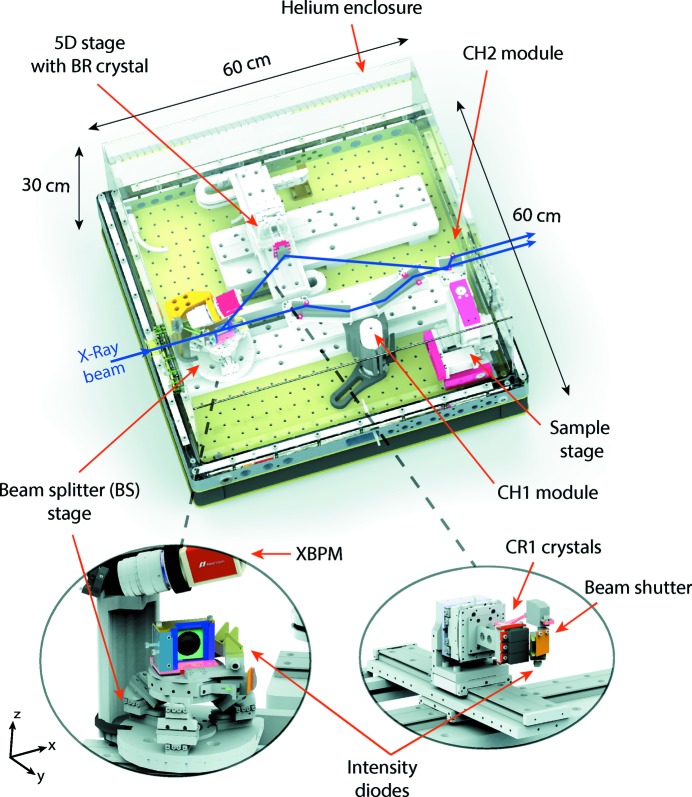
(Top) A 3D illustration of the split-and-delay system, with (bottom) detailed views of the BS and BR stages.

The device has a modular design where each optical component is placed on a separate stage, giving six degrees of freedom for the BS crystal and five for the BR crystal. Fig. 3 ▸ shows the layout of the developed system. The whole unit itself has dimensions of 60 × 60 × 30 cm with a total weight of about 60 kg. It is suitable for beamline integration directly in the experimental hutch and can be installed in the horizontal or vertical scattering plane. The device is covered with an acrylic glass enclosure, enabling operation in a helium atmosphere. This allows air absorption to be reduced and speeds up the heat flow inside the setup.

SmarAct piezo positioners were implemented for ultra-high-precision crystal alignment. The BS crystal is mounted on a movable six-axis tripod, translated along the main beam path (see Fig. 3 ▸). The BR crystal is mounted on a stage which provides three linear (*x*–*y*–*z*) and two angular motions (*r_x_*, *r_z_*). Three different crystals can be placed on this stage and selected simultaneously during operation by simply moving the crystal *z* stage (see Fig. 3 ▸). All crystal stages provide 1 nm linear and 0.03 µrad angular resolution (Table 2 ▸). In total, this gives a nominal femtosecond delay-time precision in the working energy range.

For the Case I configuration, a sample environment is designed and placed at the intersection point of the beam paths of the fixed- and variable-delay branches (see sample stage in Fig. 3 ▸). The stage consists of an *x*–*y*–*z* positioner with micrometre step resolution. Holders for various sample types, such as membranes, capillaries or any custom mounts with additional functionality, are implemented.

## 6. Alignment and diagnostics   

To monitor the intensity and shape of FEL pulses inside the delay line and determine the time between the output pulses, a set of diagnostic tools was implemented. For the setup alignment to the main beam path, an X-ray beam-position monitor (XBPM) is installed on the top of the BS stage (see Fig. 3 ▸). The monitor consists of a CMOS camera and a YAG crystal, which can be translated to the main beam path immediately in front of the beam splitter. When the YAG crystal is illuminated, the XBPM gives the 2D beam profile with 3 µm resolution. Precise alignment of the setup is achieved by translating the XBPM stage along the main beam path and tracking the position of the beam on the camera.

In order to measure the quantitative intensity values of split pulses in both branches during operation, passivated implanted planar silicon (PIPS) diodes (Owen *et al.*, 2009 ▸) are implemented in the setup. The diodes are positioned 30 mm after the BS and BR crystals and equipped with 25 µm Kapton foils. The Kapton is mounted at 45° with respect to the active area of the diode, producing the scattering signals during X-ray illumination. Shutters are installed on both branches to separate the signals at the output of the device. A splitting ratio is obtained by measuring the pulse intensities in both branches separately. The ionization chambers manufactured at DESY (Roseker *et al.*, 2009 ▸) are also used for quantitative flux measurements.

## 7. Summary   

A compact split-and-delay system for hard X-ray FEL pulses has been designed and manufactured. Delay times from −5 to 815 ps can be achieved with femtosecond resolution. This is achieved with a help of advanced piezo motors. The device can operate continuously in a wide energy range from 7 to 16 keV. Diagnostics systems such as XBPM monitors and PIPS diodes are utilized to keep track of the pulse parameters during operation.

## Figures and Tables

**Table 1:** Calculated values of the achievable delay times and expected throughput using various crystal sets τ_min_ and τ_max_ correspond to the minimum and maximum achievable delays at 7 keV. Throughput values calculated within the energy range from 7 to 16 keV. *r*_s_ represents the splitting ratio of the delayed to the main branch fractions.

	Crystal configuration							
Source Δ*E*/*E*	BS	BR	CH1	CH2	τ_min_ (ps)	τ_max_ (ps)	Throughput (%)	*r*_s_ (%)
Pink beam 10^−3^	Si(111)	Si(220)			47	179	90.9 ± 0.1	1:15
	Si(111)	Si(220)	Si(220)	Si(220)	−5	117	10.8 ± 0.8	1:1
				Si(111)				
Si(111) beam 1.4 × 10^−4^	Si(220)	Si(422)			200	815	66.3 ± 0.1	1:6

**Table 2:** Crystal stage resolutions on the different axes *r_x_*, *r_y_* and *r_z_* represent angular motions around the *x*, *y* and *z* axes, respectively.

Crystal(s)	Axis	Resolution
BS, BR	*x*, *y*, *z*	1 nm
BS, BR, CH1, CH2	*r_x_*, *r_z_*	0.03 µrad
BS	*r*_*y*_	0.03 µrad
